# A large forearm subcutaneous hematoma after contrast extravasation requires surgical managements: A case report

**DOI:** 10.1097/MD.0000000000039536

**Published:** 2024-09-06

**Authors:** Chunqiao Wu, Zhexia Jin, Yongmei Yang

**Affiliations:** a Department of Radiology, Sir Run Run Shaw Hospital, School of Medicine, Zhejiang University, Hangzhou, China.

**Keywords:** case report, computed tomography, contrast extravasation, debridement, hematoma, nursing care

## Abstract

**Rationale:**

Large extremity hematoma can rarely happen after contrast extravasation during a contrast-enhanced computed tomography scan. Some hematomas need prompt surgical managements.

**Patient concerns:**

A 77-year-old man had acute ischemic stroke and received the thrombolytic and antiplatelet therapies. He had a contrast extravasation during the computed tomography scan and developed a large hematoma in the right forearm, despite without evidence of compartment syndrome.

**Diagnosis:**

Right forearm hematoma, status post contrast extravasation.

**Interventions:**

The patient responded poorly to the routine care with arm elevation, cold pack, and wet dressing, and was finally treated by the surgical debridement, vacuum sealing drainage, fascioplasty, and skin flap repair.

**Outcomes:**

Right forearm wound healed with a scar.

**Lessons:**

Large extremity hematoma can happen after contrast extravasation during computed tomography scan, which may require surgical treatments. Careful preparation, close monitor, and prompt managements should be applied in high-risk patients.

## 1. Introduction

Computed tomography (CT) scan is a common diagnostic technique applied in the clinic. In some cases, intravenous contrast agent, most commonly with iodine-based contrast materials, is required during a contrast-enhanced CT scan.^[1]^ This process can rarely cause contrast agent extravasation. The clinical presentations after contrast extravasation varied from mild symptoms, including local discomfort, swelling, pain, numbness, and blisters, to more severe issues, such as skin ulcer, tissue necrosis, compartment syndrome, and adjacent joint involvement.^[2]^ The compartment syndrome after contrast extravasation has been well described by a number of case reports. However, large extremity hematomas can also happen after contrast extravasation, which was rarely mentioned in patients with contrast extravasation. These hematomas also requires special attention, since certain hematomas might need surgical managements in addition to routine cares.^[3]^

Here, we report 1 patient with accident contrast agent extravasation. He developed a large forearm subcutaneous hematoma that required surgical debridement and fascioplasty. We present this case, review the previous relevant literatures, and discuss the experience on this patient, with the purpose to minimize the occurrence of similar complication and improve its managements in future clinical practice.

## 2. Case presentation

On September 5, 2021, a 77-year-old man presented to the hospital due to slurry speech and right extremity weakness and numbness for 1 hour. He had a medical history of hypertension and gastrointestinal hemorrhage. Physical examination revealed muscle strength 3/5 on right extremities. Laboratory tests showed a blood glucose level of 8.5 mmol/L and activated partial thromboplastin time of 57.4 seconds. An immediate brain CT scan reported cerebral infarctions in bilateral basal ganglia and ischemic changes in the adjacent white matter. A diagnosis of acute ischemic stroke was made. He was admitted into the neurology department and received intravenous alteplase (58.5 mg) and oral aspirin (0.3 g) and clopidogrel (75 mg). Ultrasound examination showed gross intimal thickening with multiple plaque formations in the bilateral carotid arteries. A contrast-enhanced head and neck CT scan was ordered for the patient. During the examination, a 20G needle was placed in the right forearm. After confirming the blood return, contrast agent (Iohexol, Omnipague, GE Healthcare, Chicago, IL) was injected intravenously. After 30 ml of contrast agent injection, the patient developed erythema and swelling, approximately 15 × 7 cm in size, in the right forearm. There was no contrast agent observed in the CT images. A diagnosis of contrast agent extravasation was considered. The CT scan was terminated. The patient received arm elevation, ice packs, and wet dressing, with normal saline, dexamethasone, and lidocaine immediately. He was transferred back to the medical floor for close monitoring and treatment.

**Figure 1.**
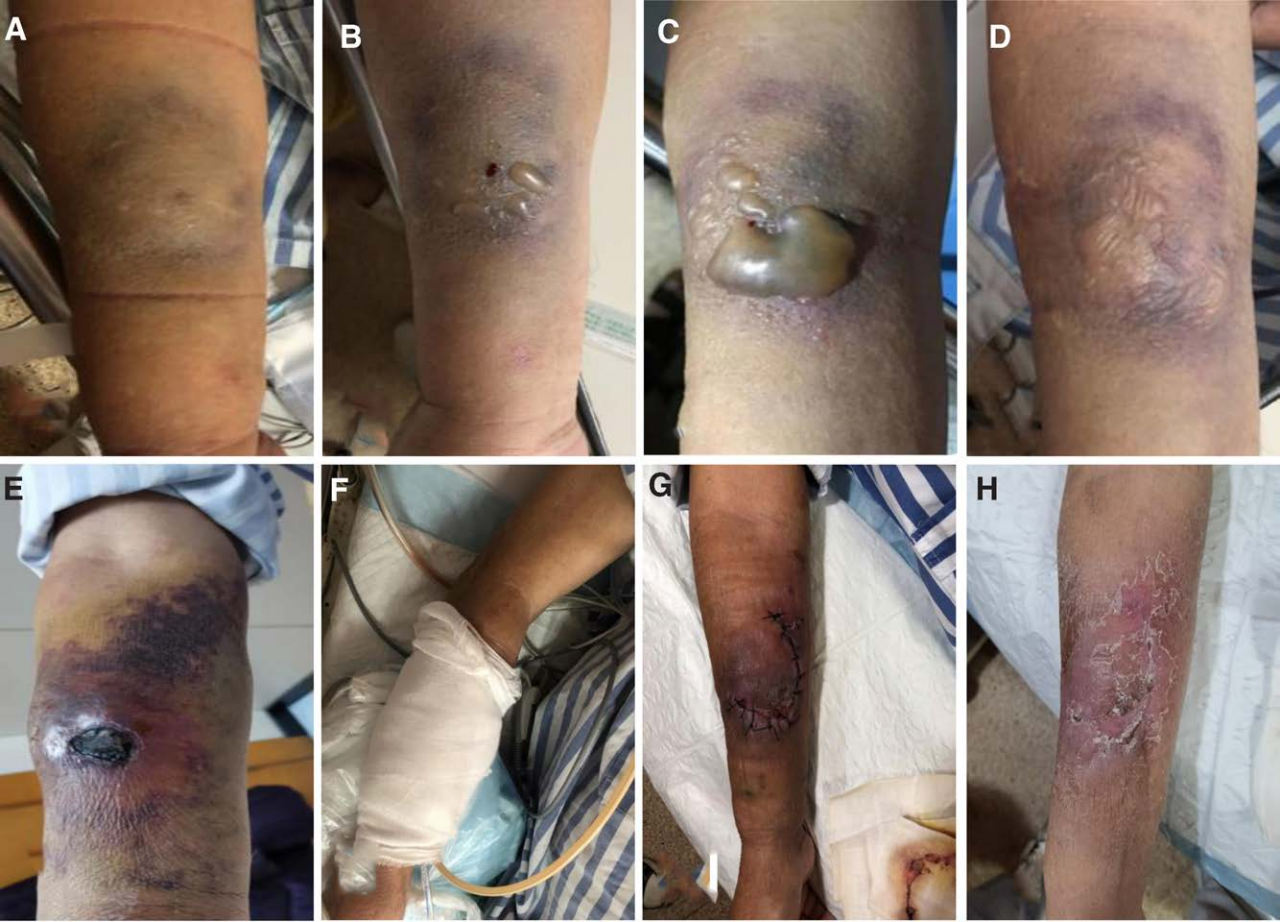
Right forearm appearance changes. (A) Right forearm swelling with ecchymosis on the first day. (B) The ecchymosis expanded with exudates and small blisters on the second day. (C) The blisters fused to form tense bullae on the third day. (D) A needle was used to aspirate the fluid in the bullae on the 4th day. (E) Swelling improved but with persistent subcutaneous hematoma. (F) After surgical debridement, the right forearm was wrapped with vacuum sealing drainage. (G) Forearm after fascioplasty. (H) Healed scar in right forearm 1 year after the surgery.

Figure 1A shows the right forearm swelling with ecchymosis on the first day. The ecchymosis expanded with exudates and small blisters on the second day (Fig. 1B). On the third day, the blisters fused to form tense bullae (Fig. 1C). On the fourth day, a needle was used to aspirate the fluid in the bullae (Fig. 1D). One week after the incident, the forearm swelling improved, with local scab. There was a skin elevation because of subcutaneous hematoma (Fig. 1E). The patient’s right hand was warm with no sign of ischemia. Ultrasound examination showed a hypoechoic area of approximately 4.9 × 4.9 × 1.7 cm, consistent with a hematoma, in the right forearm. After a multidisciplinary consultation, considering that the large size of hematoma, a decision was made to perform surgical debridement. Therefore, this patient received 2 surgical operations. The first step surgery involved skin and subcutaneous necrotic tissue excision and debridement with vacuum sealing drainage and fascioplasty under the brachial plexus block 2 weeks after the incidence of contrast extravasation (Fig. 1F). The second surgery was skin reconstruction by the adjacent skin flap 3 days later (Fig. 1G). Both steps were performed under local anesthesia by brachial plexus nerve block. The surgical operations were uneventful and the patient was discharged from the hospital. At the clinic visit 1 year after the surgery, the right forearm wound was healed with a scar (Fig. 1H). The strength of the right arm showed no difference compared with that of the left arm.

## 3. Discussions

Contrast-enhanced CT scan can be associated with complications of contrast agent extravasation. Serious complications can include compartment syndrome, with increased tissue pressure to block the circulation and perfusion to the affected extremities. The compartment syndrome after contrast extravasation has been well described by a number of case reports. However, hematoma can also happen, which was rarely reported. Here, we report a patient who developed forearm hematoma and tissue necrosis, although with no sign of compartment syndrome. He had poor response to routine care, including arm elevation, ice packs, and wet dressing, which required two-step surgical debridement and fascioplasty.

Similar case with large hematoma due to contrast extravasation that required the surgical repair was rarely reported previously. We researched the previous case reports that mentioned extremity hematoma formation after contrast extravasation (Table 1). All these previous hematomas were reported in hand and occurred simultaneously with compartment syndrome. Our patient had the hematoma occurring in the forearm. There was no sign of ischemia or neurological defects in the hand to suggest compartment syndrome. However, his hematoma responded poorly to the conservative care and finally required surgical managements. We report this unique case here to remind clinicians to not only watch for compartment syndrome but also pay attention to hematoma after contrast extravasation in high-risk patients.

**Table 1:** Case reports that mentioned hematoma formation after contrast extravasation.

Authors	Publication year	Age, sex	Location	Contrast medium	Injection volume	Clinical diagnosis	Treatments
D’ Asero, et al^[4]^	2010	80 years, female,	Hand	Nonionic, water-soluble medium	100 mL	Hematoma, compartment syndrome, bullous eruption	Fasciotomy
Belzunegui et al^[5]^	2011	50 years, female	Hand	Nonionic iodinated contrast	100 mL	Hematoma, compartment syndrome	Hematoma evacuation, fasciotomy
Yurdakul et al^[6]^	2014	60 years, male	Hand	Iohexol (nonionic)	100 mL	Hematoma, compartment syndrome	Hematoma removal, contrast aspiration, fasciotomy
Stavrakakis et al^[7]^	2018	72 years, female	Hand	Iopromide (non-iconic)	110 mL	Hematoma, compartment syndrome	Fasciotomy
Papatheodorou et al^[8]^	2022	79 years, male	Hand	Iodinated medium	n/a	Hematoma, compartment syndrome	Hematoma evacuation, fasciotomy
Current case		77 years, male	Forearm	Iohexol (nonionic)	30 mL	Hematoma	Debridement, fascioplasty, flap repair

It was reported that different factors, such as types of contrast agents, catheter size, catheterization location, injection patterns, and patient characteristics, could affect the risk of contrast extravasation. We consider that multiple reasons might contribute to the contrast agent extravasation and subsequent hematoma formation in this patient. These reasons included the following: (1) this patient received alteplase, aspirin, and clopidogrel. Al these anticoagulant and antiplatelets could certainly increase the risk of bleeding and hematoma. (2) Inappropriate arm selection for the intravenous catheter placement.^[9]^ The patient had right arm weakness and numbness because of acute ischemic stroke. His right arm had decreased sensation to pain, which delayed his perception of arm swelling from contrast agent extravasation. The selection of the arm on the healthy side might prevent contrast agent extravasation. The medical staff tend to place the intravenous needle in the antecubital veins, since veins are usually superficial and easily palpable there. However, if a patient bends the elbow, the contrast injection can be blocked, which leads to contrast extravasation. Here, we placed the intravenous needle in the volar side of right forearm, but still caused the contrast extravasation. Therefore, appropriate selection of vein for catheterization and close monitoring during the injection should applied regardless of the location of intravenous catheter placement. (3) The patient’s age (77 years old) and neurological disease (acute stroke, under thrombolytic, and antiplatelet treatments) placed him at a high risk for severe extravasation.^[10]^ Additionally, he had tiny and tortuous veins, which made it difficult to place an intravenous catheter. The nurse had to manipulate the needle, advancing and retreating the needle several times during the intravenous catheter placement, which could puncture the vessels multiple times to cause bleeding and hematoma. (4) The nurse failed to press the puncture site adequately after retreating the needle from the skin. Inadequate compression on the puncture site could increase the risk of subcutaneous bleeding and blood collection. The nurse should press not only the skin puncture site but also the surrounding site where the needle enters the vein using 2 fingers or the base of the palm to ensure adequate compression to stop bleeding. (5) The nurse failed to press the puncture site long enough to stop the bleeding. Our patient had recently received thrombolytic agent and antiplatelet therapy for acute ischemic stroke. He had a high risk of bleeding. Prolonged pressing on the puncture site should be performed to ensure the bleeding had stopped. (6) The nurse did not provide adequate education to the patient to keep his arm in the extended position. A flexed elbow might block the venous blood return and increase the intravenous pressure. During the CT scan, the increased intravenous pressure could prevent the contrast injection and cause extravasation. After contrast extravasation, the increased intravenous pressure could aggravate the bleeding from the puncture hole in the vein and cause hematoma. (7) In addition, the patient massaged the site of swelling after contrast extravasation; this could disrupt local coagulation and blood clot formation to further cause bleeding from the vessels.

Learning from this case, we propose the following special points in patients with similar clinical situations: (1) careful preparation before the venipuncture. Nurses should accurately assess, observe, and determine the distributions, as well as the thickness, depth, and elasticity, of the blood vessels in the patient. A large bore catheter (no smaller than 20G) should be used for the contrast injection. The vascular branching sites and venous valve should be avoided. Additionally, the paralytic extremity should not be used. (2) Nurses should pay special attention during the venipuncture. After successful blood return, saline flush should be attempted to ensure intravascular placement of the catheter. The patient should be instructed to lose the sleeve in the extremity and not to move the extremity with the catheter. The extremity can be temporarily immobilized with a splint, if necessary. (3) During the contrast injection, the patient should be instructed to report any discomfort in the injected arm. For patients with difficult communication, a hand-held alarm can be given to the patient to alert the providers when feeling discomfort. The contrast injection should be terminated if the injection pressure is over the limit. (4) If there is contrast extravasation, a 10 mL syringe can be connected to the catheter for attempted aspiration of any residual contrast agent, and the intravenous catheter should then be removed immediately. The ice pack can be applied. The swelling site can be covered by the wet dressing. A large tense hematoma may require aspiration, which should be performed under aseptic technique. The patient should be instructed to wear loose clothes. The extremity with the contrast extravasation should be elevated above the heart but not bent to facilitate the blood return and contrast absorption. (5) Potential complications, including contrast extravasation during the contrast-enhanced CT scan, should be fully explained to the patient and relevant family members before the procedure. Understanding the risk and complications of the procedure is important to avoid dispute and improve patient compliance and recovery. (6) Nurses should pay special attention to patients who are undergoing thrombolytic or anticoagulant treatments. These patients have a high risk for bleeding complications. If they receive venipuncture, the puncture site and surrounding area should be adequately pressed by 2 fingers or the base of the palm for an appropriate prolonged time to make sure that the bleeding has stopped. (7) Careful and repeated skin examinations should be performed. If there is any sign of subcutaneous or submucosal bleeding, such as petechiae, ecchymosis, or hematoma, or severe pain with signs of poor circulation suggesting compartment syndrome, the nurses should immediately report this to the physicians. The borders of the swelling area from the contrast extravasation should be marked. Close and prolonged follow-ups should be provided to ensure appropriate management and prevention of serious complications after contrast extravasation.

The strength of our study was that we comprehensively reviewed the nursing care on a patient who had a rare large hematoma after contrast agent extravasation. We followed this patient until 1 year after contrast agent extravasation to document his long-term prognosis. The limitation of the study was that all these experiences were from a single case. The generalizability of our nursing approach requires validation in further studies.

## 4. Conclusions

In summary, we report a rare case of subcutaneous hematoma and necrosis after contrast agent extravasation during contrast-enhanced CT scan. The patient had recently received thrombolytic and antiplatelet therapies for acute ischemic stroke, which placed him at a high risk for bleeding. He responded poorly to routine care with arm elevation, cold pack, and wet dressing and was then treated by surgical debridement, vacuum sealing drainage, fascioplasty, and skin flap repair. Appropriate nursing care, including careful pre-procedure preparation, correct puncture vein selection, close monitoring during contrast injection, cautious follow-up after injection, and frequent reevaluations are essential to minimize the risk of contrast agent extravasation and subsequent complications.

## Author contributions

**Conceptualization:** Chunqiao Wu.

**Data curation:** Chunqiao Wu, Zhexia Jin, Yongmei Yang.

**Formal analysis:** Chunqiao Wu, Zhexia Jin, Yongmei Yang.

**Methodology:** Chunqiao Wu.

**Project administration:** Chunqiao Wu.

**Supervision:** Chunqiao Wu.

**Validation:** Chunqiao Wu.

**Writing – original draft:** Chunqiao Wu, Zhexia Jin, Yongmei Yang.

**Writing – review & editing:** Zhexia Jin, Yongmei Yang.
